# Effect of Wastewater on the Composition of Bacterial Microbiota of *Phragmites australis* Used in Constructed Wetlands for Phytodepuration

**DOI:** 10.3390/plants11233210

**Published:** 2022-11-23

**Authors:** Lisa Cangioli, Maria Salobehaj, Sara Del Duca, Camilla Fagorzi, Chiara Berardi, Ester Coppini, Donatella Fibbi, Renato Fani, Alberto Vassallo

**Affiliations:** 1 Department of Biology, University of Florence, 50019 Sesto Fiorentino, Italy; 2 Center for Magnetic Resonance (CERM), 50019 Sesto Fiorentino, Italy; 3 Gestione Impianti di Depurazione Acque (G.I.D.A.) SpA, 59100 Prato, Italy; 4 School of Biosciences and Veterinary Medicine, University of Camerino, 62032 Camerino, Italy

**Keywords:** stable plant microbiota, landfill leachate, soil microbiota, wastewater treatment plant, next-generation sequencing, *Phragmites australis*, phytoremediation, constructed wetlands

## Abstract

Phytodepuration occurs in the plant-mediated remediation processes exploited to remove pollutants from wastewater, and *Phragmites australis* is one of the most used plants. This goal is achieved using constructed wetlands (CW), which are engineered systems designed to mimic the natural processes of pollutants removal. The aim of this work was to characterize the bacterial communities associated to *P. australis*, soils, and permeates of the CW of Calice (Prato, Italy), to evaluate the possible effect of wastewaters on the CW bacterial communities, through a next-generation sequencing-based approach. A total of 122 samples were collected from different tissues of *P. australis* (i.e., roots, aerial parts, and stem), soil (i.e., rhizospheric and bulk soil), and permeates, and analyzed. All samples were collected during five sampling campaigns, with the first one performed before the activation of the plant. Obtained results highlighted a specific microbiota of *P. australis*, conserved among the different plant tissues and during time, showing a lower alpha diversity than the other samples and not influenced by the more complex and variable environmental (soils and permeates) bacterial communities. These data suggest that *P. australis* is able to select and maintain a defined microbiota, a capacity that could allow the plant to survive in hostile environments, such as that of CW.

## 1. Introduction

The term phytodepuration indicates the plant-mediated remediation processes exploited to remove pollutants from water and wastewater [1]. This goal is achieved using constructed wetlands (CW), which are engineered systems designed to mimic the natural processes occurring in natural wetlands, although in a more controlled environment, to remove organic compounds, suspended solids, microbes, and heavy metals [2]. Moreover, as recently reviewed, CW can also limit the diffusion of antibiotics, antimicrobial resistance genes (ARGs), and antibiotic resistant bacteria (ARB) [1]; these are listed among the so-called “contaminants of emerging concern” (CEC), and their presence in wastewater may represent a serious danger as they can escape typical controls, spread in the environment, and even evolve [3]. Indeed, the new term “evolving CEC” (e-CEC) has been proposed for ARGs and ARB [3]. CW are recognized as a reliable technology for wastewater treatment, as well as being sustainable and cost-effective [4]; in fact, their use for the treatment of different types of wastewater has been reported, such as those from municipal, farm, and industrial sources [5]. In addition, after their use in CW, plants can potentially be used for other purposes within a circular economy framework, as their biomass results enriched of nutrients [6]: for instance, these plants could be used, at least in certain conditions, for the production of animal feedstock, fertilizers, biofuel, biochar, adsorbent, and biogas [6,7].

CW are typically classified basing on their designing parameters, such as hydrology (i.e., open water-surface flow and subsurface flow), kind of macrophyte plant growth (i.e., emergent, submerged, and free-floating), and flow path (i.e., horizontal and vertical) [2].

Plants can remove pollutants in CW through different mechanisms, such as phytoextraction (i.e., root uptake of contaminants with their accumulation in the aerial parts), phytovolatilization (i.e., pollutant uptake followed by translocation and release through the surface of stem and leaves), phytostabilization/phytoimmobilization (i.e., conversion of toxic compounds into non-toxic or less toxic forms having a reduced bioavailability), and phytodegradation (i.e., degradation of organic compounds due to enzymes contained in plant exudates) [8].

Microbial communities represent important components of CW, as they play a crucial role in the removal of pollutants. Indeed, microbes take part in the remediation process directly: for example, bacteria can help the biosorption of heavy metals by increasing their solubility and bioavailability through the reduction of soil pH and the release of chelators, and they can degrade organic compounds [8]. On the other hand, microorganisms, especially rhizospheric ones, also increase plant performances through the improvement of plant growth; for instance, they can stimulate root proliferation, protect against phytopathogens, lower the production of ethylene with their 1-aminocyclopropane-1-carboxylate (ACC) deaminases, fix nitrogen, and/or produce phytohormones such as auxin [9,10].

*Phragmites australis* (the common reed) is an aquatic emergent plant and it is one of the most used plants in phytodepuration, due to its (i) ability of removing nutrients from different kinds of wastewater, (ii) flood-tolerance, and (iii) resistance against both pollution and (iv) salinity [11,12]. Moreover, it has a wide geographical distribution, and it is particularly present in Europe and Asia [11,12].

*P. australis* is also used for phytodepuration in the pilot CW at the depuration plant in Calice (Prato, Tuscany, Italy), managed by G.I.D.A. SpA. These CW are designed for the tertiary treatment of landfill leachate (LFL) and are located downstream to a membrane bio-reactor (MBR) pretreating LFLs before their discharge in the main line of a full-scale wastewater treatment plant (WWTP) [13]. Each of the two lines of CW in Calice is a two-stage subsurface flow system (SFS), composed by a vertical system (SFS-v) followed by a horizontal one (SFS-h).

In a previous study, the bacterial culturable community associated to the roots of *P. australis* from the CW of Calice was investigated, covering a time span of 22 months [14]; one of the most interesting aspects of that study was that samples were collected both before and after the activation of the CW, allowing the analysis of the effect of wastewater influx on root-associated bacteria. This investigation highlighted how wastewater exerted a selective pressure leading to a progressive reshaping of bacterial community (e.g., 4 genera present before the activation of CW disappeared and 27 new ones colonized *P. australis* roots upon influx of wastewater). Moreover, time of exposure to wastewater was directly correlated with increasing numbers of bacterial isolates resistant to antibiotics and metal(loid)s.

The present work expands and integrates the previous one, as samples analyzed here were collected at the same timepoints [14]. While in the former work only culturable bacteria were isolated and identified, the aim here is to characterize, applying a culture-independent method, the entire bacterial communities associated to different compartments of *P. australis*, soils of CW, and permeates, through a next-generation sequencing-based investigation. In this regard, despite other similar works already available in literature, this work spans a longer time and takes in consideration not only the roots and rhizosphere of *P. australis*, which are the most studied compartments in CW, but also other anatomical compartments and soil samples collected in all the different parts of CW.

## 2. Results

### 2.1. Bacterial Diversity in Plants, Soil and Permeates Microbiome

In this work, a total of 122 samples collected from different plant compartments of *P. australis* (i.e., roots, aerial parts, and stem), soil (i.e., rhizospheric and bulk soil), and permeates were analyzed. All samples were collected during five sampling campaigns from both the vertical and the horizontal systems of CW, except for permeate samples which were not collected for the first sampling, performed before the CW activation.

DNA sequencing of V3-V4 region of 16S rDNA of these samples yielded a total of 17,736,516 reads, and 11,687,785 passed the quality filter (66% of total reads). Reads were assigned to a total of 57,921 Amplicon Sequencing Variants (ASVs) after a clustering step (Dataset S1); ASVs assigned to Archaea (25 ASVs) and ASVs not assigned to any phylum (15,076 ASVs) were removed from the following analysis.

As shown in Figure S1, all rarefaction curves reached a plateau, thus demonstrating a good representation of the microbial communities and a sufficiently deep sampling. Bacterial diversity of all samples was preserved, as shown by the Good’s coverage estimator reported in Table S1, and alpha diversity indices were estimated (Table S2).

**Figure 1:**
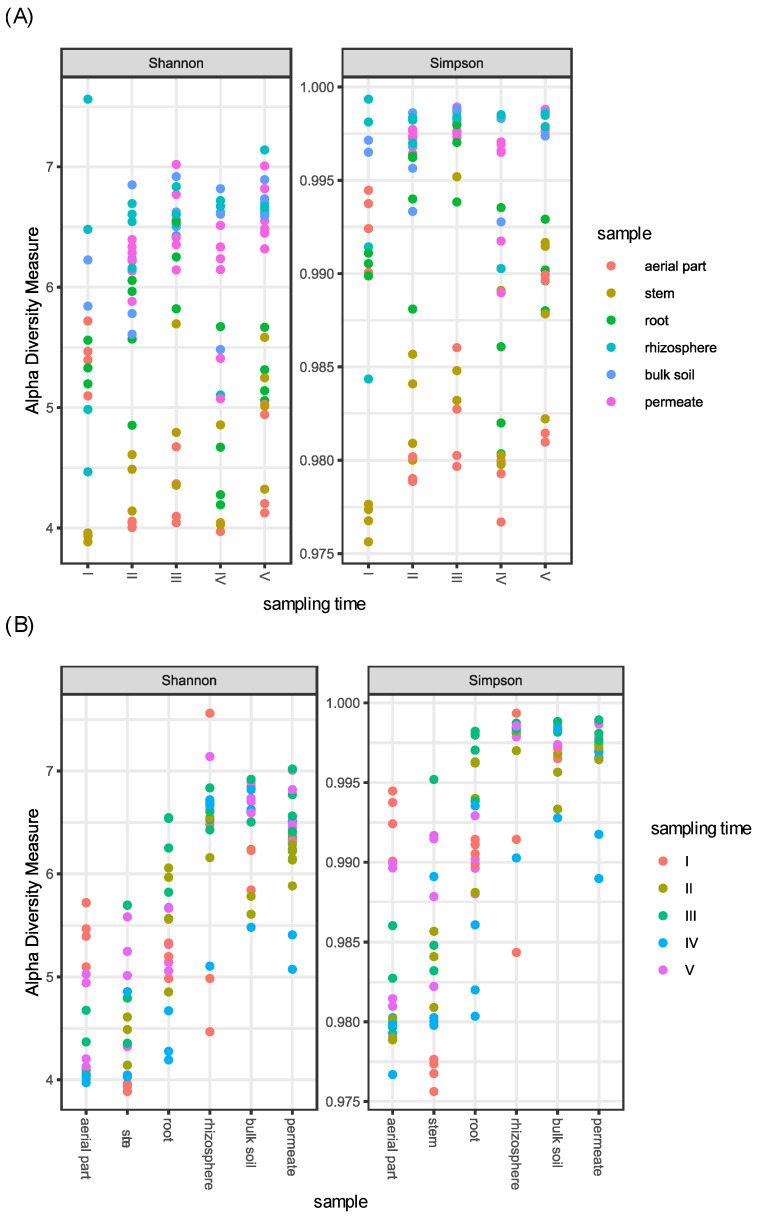
Shannon and Simpson diversity indices of the bacterial communities associated to *P. australis*, soil, and permeate samples collected in the CW during five samplings. (**A**) Samples are divided per sampling time. (**B**) Samples are divided per compartments.

Concerning the different samples, as expected, above-ground plant compartments (aerial part and stem) showed a reduced alpha diversity in comparison to soil (rhizosphere and bulk soil) and permeates, while root samples showed an intermediate bacterial diversity (Figure 1).

In particular, when compared, only (i) aerial part and stem, and (ii) rhizosphere, bulk soil, and permeates showed a non-significant difference, suggesting a similarity in the bacterial diversity of these samples. In contrast, all the other tested combinations resulted in a significant difference in alpha diversity (*p*-value < 0.001) (Table S3).

Concerning the different sampling times, (i) aerial part, stem, and root, and (ii) rhizosphere and bulk soil samples were joined in the two groups named (i) plant tissues and (ii) soils, respectively. Plant tissues differed in alpha diversity in relation to the time of sampling; in particular, the fourth sampling was different from the third and the fifth ones (*p*-value < 0.05), showing a reduction in diversity. The same difference was also observed for permeate samples, which differentiated across times according to Simpson index, indicating that the dominance of bacterial groups changes among samplings (Table S3 and Figure 1).

**Figure 2:**
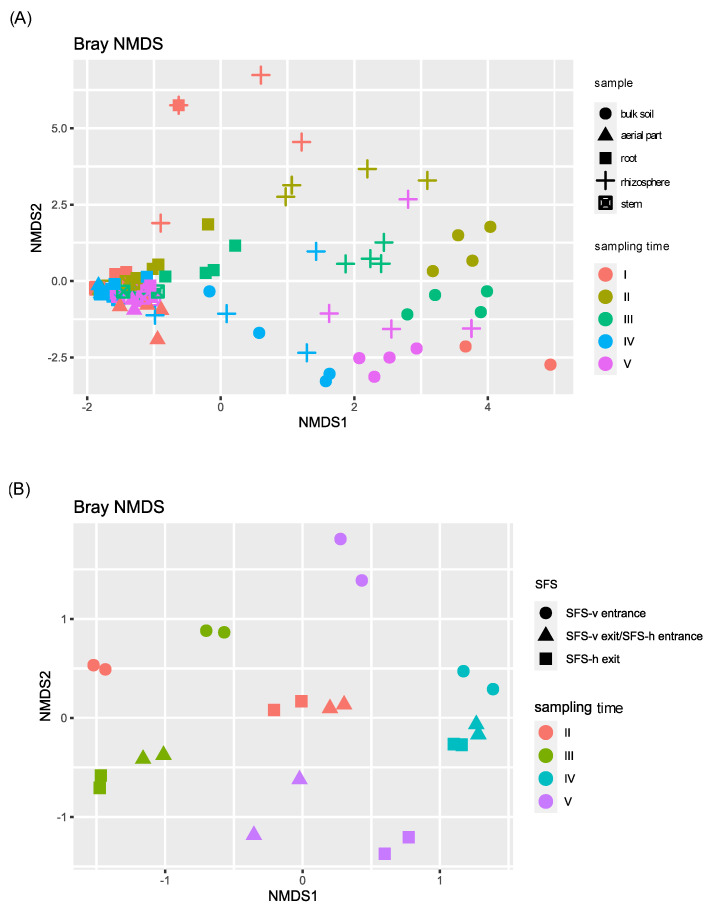
NMDS beta diversity of the bacterial communities of *P. australis*, soil and permeate samples. Samples were divided on the basis of the sampling site of the CW plant: (**A**) those collected directly from the SFS (plant tissues and soils) and (**B**) those collected from the CW irroration system (permeates).

To explore species diversity among samples, beta diversity was analyzed, performing a non-metric Multidimensional Scaling (NMDS) (Figure 2). 

Beta diversity highlighted taxonomic similarities among plant samples (aerial part, stem, root). During this time, the fourth sampling was the most different one, showing a significant *p*-value (<0.05) when compared with the first, second, and third ones (Table S4). On the contrary, soil and permeate bacterial communities were highly different from each other and from plant tissues samples, and deeply influenced by the time of sampling, as confirmed by the PERMANOVA analysis reported in Table S4. In contrast, no differences were observed considering the two CW systems.

### 2.2. Taxonomic Composition of Plant, Soil, and Permeate Bacterial Communities

Total ASVs were classified into 1019 genera belonging to 50 phyla, revealing that more than 80% of the ASVs were classified into six phyla, i.e., Proteobacteria (36.78%), Bacteroidota (17.77%), Actinobacteriota (7.49%), Patescibacteria (7.29%), Planctomycetota (5.82%), and Verrucomicrobiota (5.6%). The lowest number of phyla was detected in the aerial part (15 phyla) and stem (20) of the plants, while the highest number was detected in permeate samples, in which 46 different phyla were observed. In all the compartments, the most frequent phyla were Proteobacteria (comprised between 31.6% and 54.2%) and Bacteroidota (between 13.2% and 24.9%). The third most frequent detected phylum was Firmicutes for the aerial part and the stem of *P. australis* (with frequencies of 10% and 7.8%, respectively), Planctomycetota for the roots (6.9%), Patescibacteria for the rhizosphere (8.1%), Actinobacteriota for the bulk soil (12.2%), and Patescibacteria for the permeates (9.8%).

**Figure 3:**
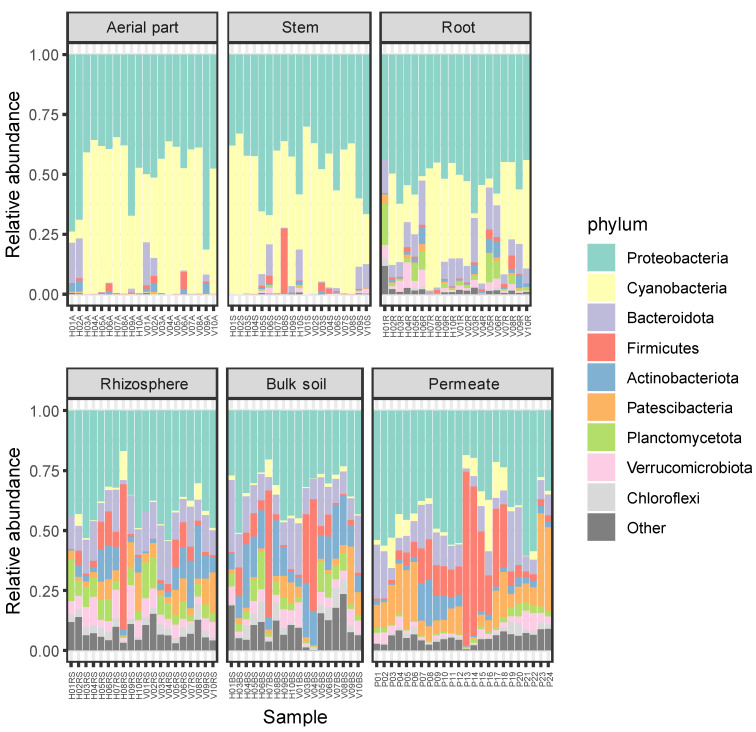
Relative abundances of bacterial phyla in each sample. ASVs representing < 5% of the whole community have been reported as “Other”.

The relative abundances of detected phyla are reported in Figure 3, where they appear to be more similar with one another among the plant tissues (i.e., aerial part, stem, and root), showing higher relative abundances for Proteobacteria and Cyanobacteria. On the contrary, soil and permeate samples showed higher levels of diversity, in agreement with results regarding alpha diversity reported in Section 2.1.

### 2.3. Effect of the Different Sampling Times on the Taxonomic Composition of Permeates Microbiome

In this study, permeate represents the main external factor acting on the CW plant components; thus, we focused our attention on permeate microbial composition. The limited number of collected permeate samples did not allow us to perform statistical analyses for the three different sampling sites of the CW. For this reason, all the permeate samples collected at the same timepoint were treated as a unique group regardless of their spatial provenience to focus only on differences due to the time of sampling.

**Figure 4:**
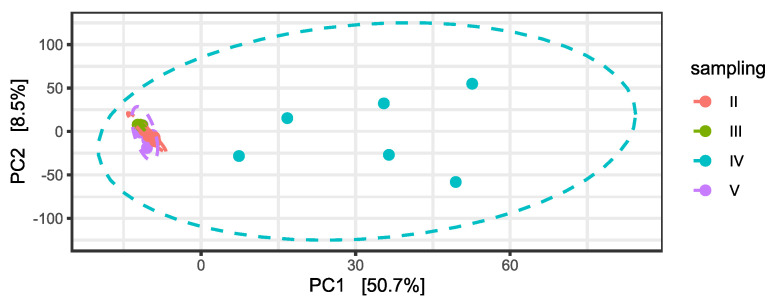
Principal Component Analysis (PCA) on centered log-ratio transformed counts at ASV level and variance percentage explained by each component. Ellipses show the Normal-theory confidence regions with alpha = 0.95. Permeate samples are colored in relation to the time of sampling.

Subsequently, differential abundance analysis was performed on permeates samples during time. Differences between permeates were statistically significant, particularly in relation to the fourth and the fifth samplings, as confirmed by a PERMANOVA on the PCA to the centered log-ratio (Table S5 and Figure 4).

Differences in permeate taxonomic composition are present in relation to 197 ASVs with statistically different abundance when considering sampling timepoint (Table S6). Most of the 197 ASVs include members of the orders Flavobacteriales, Sphingomonadales, Rhodobacterales, Oceanospirillales, and Pseudomonadales.

### 2.4. Prediction of Potential Functions and Metabolic Pathways of Permeates

**Figure 5:**
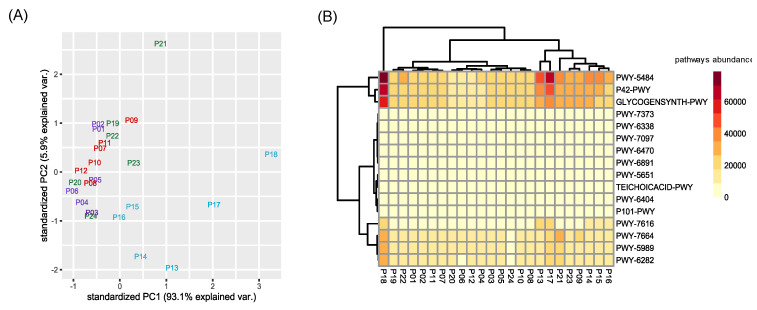
Clustering of pathways abundance. (**A**) Principal Component Analysis (PCA) was performed considering the predicted functional abundances of all identified pathways. In violet permeates of the second sampling, in red for the third, in blue for the fourth, and in green for the fifth. (**B**) Heatmap considering only the top three pathways that differentiate each contrast between varieties returned by SIMPER test. Codes of represented pathways refer to the MetaCyc Metabolic Pathway Database.

Functional profiles of the permeate samples microbiota were then investigated. All the identified metabolic pathways present in the taxa forming the microbiota are listed in Table S7. We identified a total of 423 pathways, all distributed in all samples. However, pathway abundances showed a clustering according to sampling time, in particular for the fourth sampling (Figure 5A). 

Using a SIMPER test, we selected the top three pathways that mostly differentiate the four sampling timepoints (in terms of amount of explained variance). For each contrast between different sampling times, the three most represented metabolic pathways were selected, for a total of sixteen different pathways (i.e., PWY-6470, PWY-7373, PWY-7664, P42-PWY, GLYCOGENSYNTH-PWY, PWY-5484, PWY-6891, PWY-6404, P101-PWY, PWY-5651, TEICHOICACID-PWY, PWY-5989, PWY-6282, PWY-6338, PWY-7097, PWY-7616), related to the (i) metabolism of aerobic respiration, (ii) antibiotic resistance, (iii) vanillin and vanillate metabolism, and (iv) biosynthesis of stress related compounds (Table S8). The clustering of this subset of pathways agreed with the overall results of the taxonomic diversity observed for permeate samples (Figure 5A). However, a heatmap reporting the sixteen most expressed pathways did not show a particular clustering of permeate samples, with the exception of those belonging to the fourth sampling, which grouped together (Figure 5B).

## 3. Discussion

The aim of this work was the characterization of the whole bacterial communities associated to different compartments of the plant *P. australis*, soils, and permeates of the pilot constructed wetlands (CW) of Calice (Prato, Italy), for a total of 122 samples, to evaluate the possible effect of wastewaters on the CW bacterial communities. The composition of the total microbiota was determined through five samplings spanning from March 2017 to December 2018, with the first one performed before the activation of the plant, thus, before the entry of wastewaters.

The analysis of the diversity within each sample highlighted a higher diversity for soils (i.e., rhizosphere and bulk soil) and permeates with respect to the plant tissues (i.e., aerial part, stem, and root). Bacterial communities’ compositions were similar for the different plant compartments, while differences were observed for soils and permeates, which were highly different from each other and from plant tissues samples, showing a deep dependence on the sampling time.

This result is a quite interesting aspect. Indeed, permeates continuously flow through CW, staying for relatively short times inside the tanks (in a time range spanning from few hours to few days, depending on the season, weather, etc.). On the contrary, *P. australis* plant, its associated soil, and the surrounding soil filling the tanks of CW, remain stable in the CW and are subjected to a slower renewal. It is unlikely that permeate could shape CW total microbiota completely; nonetheless, it is very probable that permeates somehow affect CW bacterial communities. The fact that soils and permeates bacterial communities resulted different from each other, despite their strict physical interaction, can be explained based on the continuous influx of different wastewaters, whose differences can be due to seasonal changes and/or origin, potentially leading even to remarkable variations. However, the effect of permeates on environmental microbiota can be highlighted by the similarity in bacterial diversity among these samples. On the other hand, *P. australis* bacterial community seems to remain stable among the different plant tissues, with minor variations along time. This is also true in the case of *P. australis* roots, which are in close contact with the environment. This aspect allows the hypothesis that *P. australis* is able to select and hold a specific microbiota, as already proposed in the case of other plants such as medicinal ones [15]. Indeed, it was previously reported that, as in the case of wastewater treatment plants, interactions between bacteria exert the main effect in determining the composition of the bacterial community in comparison to environmental properties [16,17]. Moreover, maintenance of a stable balance between host and associated microorganisms could improve plant health through prevention of dysbiosis and pathogen colonization [18]. All these considerations are particularly fitting in the case of *P. australis* growing in CW: in fact, presence of a stable microbiota could also be related to its ability to thrive in a harsh environment, characterized by the presence of diverse pollutants of various origins. From a biotechnological and applicative viewpoint, stability of the microbiota associated to the plant tissues could be exploited to shape and improve phytoremediative performances of CW. Indeed, *P. australis* could be inoculated with selected beneficial bacterial strains before its implant in CW, possibly leading to a selective advantage for those specimens having this controlled microbial flora. On the other hand, regular checks of the microbial community might be useful to predict an upcoming decrease in remediative performance due, for instance, to dysbiosis events. However, new experiments are certainly needed to confirm these hypotheses on the field, and comparisons between the bacterial communities of *P. australis* from different CW worldwide could represent an untapped source of valuable information to improve phytodepuration efficiency.

No evident shift of the microbial communities seems associated to seasonal changes and/or meteorological factors, reinforcing the hypothesis that the permeate acts as an exogenous factor influencing changes mainly in soil microbial communities without however replicating its own composition. Under the light of possible functional differences among the bacterial communities present in the permeate, PICRUSt2 results mirrored, in part, the taxonomic profiling of permeate samples. Pathways related to antibiotic resistance were found in relation with the taxonomy of the bacterial communities associated to the permeates. This is in line with the provenience of the wastes treated in the G.I.D.A. SpA plant, designed for the tertiary treatment of landfill leachate (LFL), thus presenting material from anthropogenic provenience and potentially rich in antimicrobial compounds [19]. Natural vanillin production, evidenced by the presence of the PWY-6338 and PWY-7097 pathways, represents an important perspective to exploit the potential of bacterial communities associated to waste materials. The exploitation of biological synthetic pathways, such as those involving microorganisms, rather than relying on petroleum derivatives as major raw materials, can result in an environmentally friendly process [20].

In particular, permeates harvested during the fourth timepoint grouped separately from the others, similar to the pattern shown in the PCA analysis of ASVs (Figure 4). Interestingly, the metabolic pathways, which mostly differentiated the permeate collected during the fourth sampling from the others, were mainly related to an increased aerobic respiration and fatty acid biosynthesis (PWY-7664, PWY-5989, PWY-6282). These permeate bacterial communities contained a higher amount of Firmicutes, a phylum including several aerobic bacterial groups thriving in soil, such as *Bacillus*. Fatty acid biosynthesis has already been described in numerous studies on CW [21,22,23]. Hydrolysis, acidification, and solubilization of complex organic compounds are extremely influenced by environmental factors such as temperature and hydraulic retention time [24,25]. For this reason, it is possible to observe fluctuation of this pathway, since the G.I.D.A. SpA CW plant is subjected to this kind of fluctuations. Finally, pathways PWY-7616 and P42-PWY are linked to the methanol metabolism. Indeed, enzymes of an incomplete reductive TCA cycle have been experimentally demonstrated as present in the autotrophic methanogen *Methanococcus maripaludis* [26].

In conclusion, this work allowed a further characterization of the bacterial community associated to *P. australis* and its surrounding environment exploited for phytodepuration in CW. Previous results obtained on the culturable component of *P. australis* root microbiota showed that resistance against antibiotics and metal(loid)s increased in relation to the time of exposure to wastewater. Here, we observed the main changes regarding environmental samples, and that plants host a more stable bacterial community. Since analysis of the functional profiles of permeate sample microbiota revealed the presence of pathways related to antibiotic resistance, it cannot, a priori, be excluded the occurrence of horizontal gene transfer events between wastewater- and plant-associated bacteria.

## 4. Materials and Methods

### 4.1. Site Description and Samplings

Samples of *P. australis*, soils, and permeates were collected from the CW within the wastewater treatment plant in Calice (Tuscany, Italy). The site and CW were previously described [13,14]. Briefly, the CW plant consists of two independent lines, namely, “Lane A” and “Lane B”, each representing a two-stage subsurface flow system (SFS), with a vertical system (SFS-v) followed by a horizontal one (SFS-h). Samples were collected during five sampling campaigns spanning 22 months (i.e., first—March 2017; second—July 2017; third—November 2017; fourth—June 2018; fifth—December 2018), with the first one performed before the activation of the CW (i.e., no permeates and LFLs were present yet). Samples were harvested using sterile plastic bags and transferred immediately to the laboratory for DNA extraction. Three plant and soil samples collected from each SFS were pooled before proceeding with DNA extraction. Three kinds of permeates were sampled, corresponding to three different points of the CW: entrance of the SFS-v, exit from the SFS-v (i.e., entrance of the SFS-h), and exit from the SFS-h. Each sample was collected and analyzed in duplicate. Except for permeates, all samples were name-coded based on (i) the SFS of origin (i.e., “V” for SFS-v and “H” for SFS-h), (ii) the replicate (1–2, 3–4, 5–6, 7–8, and 9–10 for the first, second, third, fourth, and fifth sampling, respectively), and (iii) the kind of sample (i.e., “A”, “S”, “R”, “RS”, and “BS” for aerial part, stem, root, rhizospheric soil, and bulk soil, respectively) (Table 1). Permeates follow a progressive nomenclature based on (i) the sampling point and (ii) timing of sampling (Table 1).

### 4.2. eDNA Extraction and 16S rRNA Gene Amplicon Sequencing

Environmental DNA (eDNA) extraction was performed using the PowerLyzer PowerSoil DNA Isolation Kit (MO BIO Laboratories), according to the protocol provided by the manufacturer, under sterile conditions. Quality of DNA was assessed by 0.8% w/v agarose gel electrophoresis. Library construction and DNA sequencing of V3-V4 region of 16S rDNA were performed by IGA Technology Services (Udine, Italy). The MiSeq Illumina platform was used for DNA sequencing in the 2 × 250 paired-end format. All the sequenced reads were submitted to Sequence Read Archive (SRA) database under the accession number PRJNA885383.

### 4.3. Bioinformatic and Statistical Analyses

Illumina reads were trimmed using ‘Cutadapt’ (version 3.7) [27]. Paired-end sequences were clustered into Amplicon Sequence Variants (ASVs) following the default settings of the DADA2 pipeline (version 1.16) reported at https://benjjneb.github.io/dada2/tutorial.html (accessed on 12 April 2022) [28], relying on the R environment (version 4.0.5) [29]. After the quality check, reads were filtered using the DADA2 ‘filterAndTrim’ function, setting the maximum error rate to 2 and the fixed lengths to 250 and 220 bp for forward and reverse reads, respectively. Trimmed sequences were the input for error rate estimation using ‘learnErrors’ function with default parameters. Following denoising and inferring variants, sequences were merged [30]. After removing chimeras, taxonomic annotation was performed using the Silva database version 132 [31]. The obtained differential abundance tables were analyzed with the ‘phyloseq’ R package (version 1.34.0) [32]. ASV assigned to mitochondria and chloroplasts and those not assigned to Bacteria were removed.

Statistical tests on microbial communities were performed in R environment (version 4.0.5). Rarefaction curves were obtained using the function ‘ggrare()’ within ‘ranacapa’ R package (version 0.1.0) [33]. For alpha diversity analysis, the different indices were calculated and plotted using the function ‘estimate_richness()’ within ‘phyloseq’ R package (version 1.34.0) [34]. A Wilcoxon test for multiple comparison of averages was performed on alpha diversity indices (Shannon and Simpson) using the ‘pairwise.wilcox.test()’ function within the ‘stats’ R package (version 4.0.5). Good’s coverage was calculated through the R functions ‘goods()’ within the ‘QsRutils’ R package (version 0.1.5) [35].

Beta-diversity was analyzed using the ‘adonis()’ function within the ‘vegan’ R package (version 2.5-7) [36]. Distances were calculated using the ‘ordinate()’ function within the ‘phyloseq’ R package (version 1.34.0) with ‘bray’ as distance and ‘NMDS’ as method settings [32]. Plots were built using the ‘plot_ordination()’ function within the ‘phyloseq’ R package (version 1.34.0). To assess statistical significance, the function ‘pairwise.adonis()’ within the ‘pairwiseAdonis’ R package (version 0.0.1) was used. Using the ‘ggplot()’ function within the ‘ggplot2’ R package (version 3.3.5) [37], plots were built. Differential abundance analysis was performed using the R package DeSeq2 v1.30.1 to identify the ASVs/taxa differentially expressed in the samples. The functions ‘phyloseq_to_deseq2()’, ‘DESeq()’ and ‘results()’ were used [38].

### 4.4. Prediction of Functional Abundances

In order to investigate and predict the functional potential for each determined sequence in microbial communities, the software PICRUSt2 (Phylogenetic Investigation of Communities by Reconstruction of Unobserved States, https://github.com/picrust/picrust2, accessed on 12 April 2022) was used. The metabolic pathway abundances and functional gene profiling were obtained by comparison with MetaCyc and Kyoto Encyclopedia of Genes and Genomes (KEGG) databases [39]. The most relevant pathways for SFS differentiation were ranked running a SIMPER test using the function ‘simper()’ with the ‘vegan’ R package (version 2.5.7). For each contrast between time of sampling, the top three differentially expressed pathways were selected and analyses were carried out using the functions ‘prcomp()’ to perform a Principal Component Analysis (PCA) and ‘heatmap()’ in ‘stats’ R package (version 4.0.5) to graphically represent results.

## Figures and Tables

**Table 1:** Description of the samples collected in this work, and associated identification codes.

SFS	Sample	Sampling				
		I	II	III	IV	V
SFS-v Entrance	Permeate		P1	P7	P13	P19
			P2	P8	P14	P20
SFS-v	Aerial part	V01A	V03A	V05A	V07A	V09A
		V02A	V04A	V06A	V08A	V10A
	Stem	V01S	V03S	V05S	V07S	V09S
		V02S	V04S	V06S	V08S	V10S
	Root	V01R	V03R	V05R	V07R	V09R
		V02R	V04R	V06R	V08R	V10R
	Rhizosphere	V01RS	V03RS	V05RS	V07RS	V09RS
		V02RS	V04RS	V06RS	V08RS	V10RS
	Bulk soil	V01BS	V03BS	V05BS	V07BS	V09BS
		V02BS	V04BS	V06BS	V08BS	V10BS
SFS-v Exit SFS-h Entrance	Permeate		P3	P9	P15	P21
			P4	P10	P16	P22
SFS-h	Aerial part	H01A	H03A	H05A	H07A	H09A
		H02A	H04A	H06A	H08A	H10A
	Stem	H01S	H03S	H05S	H07S	H09S
		H02S	H04S	H06S	H08S	H10S
	Root	H01R	H03R	H05R	H07R	H09R
		H02R	H04R	H06R	H08R	H10R
	Rhizosphere	H01RS	H03RS	H05RS	H07RS	H09RS
		H02RS	H04RS	H06RS	H08RS	H10RS
	Bulk soil	H01BS	H03BS	H05BS	H07BS	H09BS
		H02BS	H04BS	H06BS	H08BS	H10BS
SFS-h Exit	Permeate		P5	P11	P17	P23
			P6	P12	P18	P24

## Data Availability

All sequences have been submitted online. Metagenomic sequences were deposited in the NCBI Sequence Read Archive (SRA) under the accession PRJNA885383. All the scripts used for the analyses are available at https://github.com/lisacangioli/Phragmites-australis.git (accessed on 29 September 2022).

## Supplementary Materials

The following supporting information can be downloaded at: https://www.mdpi.com/article/10.3390/plants11233210/s1, Dataset S1: total detected ASVs; Figure S1: rarefaction curves; Table S1: Good’s coverage; Table S2: alpha diversity indices; Table S3: ANOVA on the alpha diversity measures; Table S4: PERMANOVA on the beta diversity measures; Table S5: PERMANOVA analysis of clr transformed permeate samples, Table S6: differentially abundant ASVs in permeate samples; Table S7: identified metabolic pathways; Table S8: pathways differentiating each contrast between varieties.
